# Characterizing Neurocardiovascular Responses to an Active Stand Test in Older Women: A Pilot Study Using Functional Data Analysis

**DOI:** 10.3390/s25123616

**Published:** 2025-06-09

**Authors:** Feng Xue, Roman Romero-Ortuno

**Affiliations:** 1 Discipline of Medical Gerontology, School of Medicine, Trinity College Dublin, D02 PN40 Dublin, Ireland; romeroor@tcd.ie; 2 Falls and Syncope Unit, Mercer’s Institute for Successful Ageing, St James’s Hospital, D08 NHY1 Dublin, Ireland

**Keywords:** active stand test, functional data analysis, neurovascular response, cardiovascular monitoring, frailty, initial orthostatic hypotension, BMI, NIRS, photoplethysmography, aging

## Abstract

This observational pilot study investigated neurocardiovascular responses to an active stand test using continuous physiological monitoring and functional data analysis (FDA) in older women. A sample of 25 community-dwelling female adults aged 59–78 years (mean age: 70.3 years) participated. Participants were dichotomized into comparison groups based on five factors: age (<70 vs. ≥70 years); the presence of initial orthostatic hypotension (IOH, yes/no); body mass index (BMI < 25 vs. ≥25 kg/m^2^); antihypertensive medication use (yes/no); and physical frailty status assessed by the Survey of Health, Ageing and Retirement in Europe—Frailty Instrument (SHARE-FI score < −0.5 vs. ≥−0.5). Each participant completed an active stand test during which six physiological signals were continuously recorded: systolic (sBP) and diastolic (dBP) blood pressure and heart rate (HR) via digital artery photoplethysmography and left frontal oxygenated hemoglobin (O_2_Hb), deoxygenated hemoglobin (HHb), and tissue saturation index (TSI) via near-infrared spectroscopy (NIRS). The signal analysis focused on a standardized 200 s window spanning 50 s before to 150 s after the stand, with all signals resampled and synchronized at 5 Hz. FDA was used to statistically compare the full time series between groups for each signal. Group-level differences revealed that younger participants (<70 years) exhibited significantly higher HR in multiple periods following the stand (~10 s, ~30 s, ~90 s, and ~140 s post-stand) compared to their older counterparts. Participants with IOH demonstrated significantly lower sBP at ~10 s, ~80 s, and ~130 s post-stand and lower dBP at ~10 s post-stand. Among participants classified as overweight/obese (BMI ≥ 25 kg/m^2^), significantly lower levels of HHb were observed at ~10 s, ~30–50 s, and ~60 s post-stand, while O_2_Hb levels were reduced at ~50 s, ~60 s, ~70–110 s, ~130 s, and ~140 s post-stand. No statistically significant group-level differences were observed based on antihypertensive medication use or frailty status. These findings demonstrate the utility of FDA in detecting subtle, time-dependent physiological variations during orthostatic challenge and underscore the value of continuous neurocardiovascular monitoring in assessing orthostatic tolerance in aging populations.

## 1. Introduction

The ability to maintain blood pressure and cerebral perfusion upon standing is a critical function of the body’s neurocardiovascular systems. In older adults, even subtle impairments in these regulatory mechanisms can lead to orthostatic intolerance, characterized by transient hypotension; dizziness; falls; and, in some cases, loss of consciousness [1,2]. These symptoms often occur in the context of underlying age-related changes in vascular compliance, baroreflex sensitivity, and cerebral autoregulation and may be exacerbated by comorbidities such as obesity, antihypertensive medication use, and frailty [3,4,5].

The active stand test is a well-established clinical tool for evaluating orthostatic responses. Traditional interpretations typically rely on point estimates or averaged intervals of physiological data, which may overlook brief but clinically meaningful deviations that occur in the seconds immediately following postural change [6]. This is particularly relevant in detecting initial orthostatic hypotension (IOH), a transient and underdiagnosed form of orthostatic instability associated with adverse outcomes in older adults [7,8].

Recent advances in sensor technologies allow for the continuous collection of cardiovascular and cerebral hemodynamic signals with high temporal resolution. These include beat-to-beat blood pressure and heart rate measurements via photoplethysmography, as well as cerebral oxygenation assessments using near-infrared spectroscopy (NIRS) [9]. While statistical parametric mapping (SPM) has proven useful in large epidemiological datasets for analyzing time series data [10], its application in smaller clinical samples is limited by reduced statistical power and the requirement of explicit spatial smoothing to meet the assumptions of random field theory used for statistical inference. In such contexts, conventional statistical approaches may struggle to detect subtle or time-specific group differences in physiological signals.

Functional data analysis (FDA) offers a flexible alternative by enabling statistical comparisons across entire signal trajectories. Unlike traditional methods that rely on discrete time points or summary measures, FDA models the continuous nature of physiological responses, making it particularly well suited for capturing transient differences in small-sample studies. This approach has seen growing use in medicine and neuroscience research, where dynamic regulatory processes are often of primary interest [11].

In this pilot study, we applied FDA to investigate neurocardiovascular responses to an active stand test in a cohort of community-dwelling older women. Using continuous recordings of blood pressure, heart rate, and cerebral NIRS, we examined whether specific clinical characteristics—older age, the presence of IOH, higher body mass index (BMI), antihypertensive medication use, and higher physical frailty—were associated with distinct temporal patterns in physiological responses to the orthostatic challenge.

## 2. Materials and Methods

### 2.1. Study Sample

This study involved a small sample of volunteer community-dwelling older women, recruited between May and July 2023 from a local healthy aging community group in South Dublin, Ireland. Eligibility criteria included age ≥ 50 years, the ability to provide written informed consent, independent mobility (with or without a walking aid), and being able to transfer from lying to standing independently or with only minimal assistance. The exclusion criterion was the presence of an indwelling electronic device (e.g., a pacemaker).

Participants were invited to attend a dedicated research assessment at the Falls and Syncope Unit, Mercer’s Institute for Successful Ageing (MISA), St. James’s Hospital, Dublin. The full research setup has been described in detail elsewhere [12]. Ethical approval was obtained, all participants provided written informed consent, and the study was conducted in accordance with the principles of the Declaration of Helsinki.

### 2.2. Participant Characteristics

For the comparison of continuous orthostatic physiological responses, the sample was dichotomously classified based on age, presence of IOH, body mass index (BMI), antihypertensive medication use, and physical frailty status. Participants were grouped by age using a threshold of 70 years (younger: <70; older: ≥70). IOH was defined based on hemodynamic criteria—regardless of reported orthostatic symptoms—as a transient drop in systolic blood pressure (≥40 mmHg) or diastolic blood pressure (≥20 mmHg) within 15 s of standing [7], measured during the active stand test. BMI was used to categorize participants as overweight (≥25 kg/m^2^) or obese (≥30 kg/m^2^) (vs. non-overweight), in accordance with World Health Organization (WHO) standards [13]. Antihypertensive use was determined based on self-reported current medication and classified according to the WHO Anatomical Therapeutic Chemical (ATC) classification system. This included medications in the following ATC classes: C02 (antihypertensives), C03 (diuretics), C07 (beta-blockers), C08 (calcium channel blockers), and C09 (agents acting on the renin–angiotensin system) [12]. Higher physical frailty was defined using the continuous scoring system of the Survey of Health, Ageing and Retirement in Europe—Frailty Instrument (SHARE-FI) score [14], with a threshold based on the sample median.

### 2.3. Active Stand

Participants underwent an active stand test in a quiet, temperature-controlled room (maintained at 21–23 °C). Prior to standing, each participant rested in a supine position for approximately 5 min while the Finapres^®^ Nova device was calibrated using oscillometric brachial measurements. This calibration ensured accurate beat-to-beat blood pressure monitoring via a finger cuff on the left hand, with continuous signal adjustment using the PhysioCal function and the height correction unit. Following supine rest and calibration, participants were instructed to stand up promptly—unaided unless minimal assistance was required—and to remain standing for 3 min. Throughout the entire procedure, continuous recordings of cardiovascular and neurovascular signals were collected. Cardiovascular signals included beat-to-beat systolic (sBP) and diastolic (dBP) blood pressure and heart rate (HR), captured via digital photoplethysmography. Neurovascular signals included frontal cerebral oxygenation parameters—oxygenated hemoglobin (O_2_Hb), deoxygenated hemoglobin (HHb), and tissue saturation index (TSI)—measured using near-infrared spectroscopy (NIRS). Full details on the active stand protocol are reported elsewhere [12].

### 2.4. Instrumentation

#### 2.4.1. Continuous Cardiovascular Signals

A Finometer device (Finometer MIDI, Finapres^®^ Medical Systems, Amsterdam, the Netherlands) was used to noninvasively monitor the reconstructed arterial pressure on a beat-to-beat basis. This photoplethysmography-based device records the pressure waveform of the digital arteries at a sampling rate of 200 Hz using the volume-clamp method. The volume of the finger artery, detected by optical sensors embedded in the finger cuff, is kept constant throughout the assessment via a pneumatic control system that dynamically adjusts cuff pressure [15]. Importantly, the volume-clamp method has shown strong agreement with both intra-arterial blood pressure monitoring [16] and the auscultatory method [17]. The Finometer also accounts for hydrostatic pressure differences between the finger and heart level using a height correction unit, which includes a position sensor mounted to the finger.

#### 2.4.2. Continuous Neurovascular Signals

NIRS is a non-invasive, non-ionizing optical technique widely employed for monitoring changes in oxygenated and deoxygenated hemoglobin concentrations across various human tissues [18,19,20]. Previous studies have demonstrated that NIRS readings are consistent with other measurement modalities in various applications, such as cerebral blood flow [21] and skeletal muscle contractions [22]. NIRS’ versatility and high temporal resolution, facilitated by capabilities in time-resolved, frequency-domain, and continuous wave spectroscopic implementations, render its potential for a wide range of applications in both research and clinical settings [23]. While direct measurements, such as the absorption near 850 nm that reflects oxygenated hemoglobin (O_2_Hb) and the absorption around 760 nm that indicates deoxygenated hemoglobin (HHb), are routinely reported, derived indices such as the tissue saturation index (TSI), typically calculated as TSI = 100 × O_2_Hb/(O_2_Hb + HHb) [24], are also commonly used.

A wireless NIRS device, the PortaLite^®^ (Artinis Medical Systems, Elst, the Netherlands), was used to measure O_2_Hb, HHb, and TSI signals via a relative concentration method based on the Beer–Lambert Law. With an optical sensor comprising an emitter and three long-range receivers, the PortaLite^®^ has the ability to transmit multi-channel, real-time data through Bluetooth^®^ at a maximum sampling frequency of 50 Hz. The user interface for the setup, recording, and export of NIRS data was accommodated by Oxysoft v3.0.53. The NIRS sensor was affixed approximately 2 cm above the left eye (approximately the FP1 (left frontal) position of the 10 to 20 electrode system (3 cm lateral and 3.5 cm superior to the nasion) [25], and the sampling frequency was set to 50 Hz for all recordings. The noise caused by the ambient light was minimized and kept consistent via a black headband covering the sensor.

### 2.5. Signal Acquisition and Synchronization

This study focused on a standardized 200 s segment of the active stand test, spanning 50 s before to 150 s after the initiation of standing. Beat-to-beat cardiovascular signals acquired from the Finapres^®^ MIDI device were interpolated to 5 Hz. Neurovascular signals recorded by the PortaLite^®^ NIRS device were downsampled to the same frequency (5 Hz) for consistency. Signal synchronization was achieved using multiple manual event markers inserted throughout the recordings. The precise onset of the stand—defined as the moment participants began transitioning from supine to standing—was identified by triggering a keyboard event marker labeled “active stand”. This neurovascular synchronization followed the same methodology as described elsewhere [9].

### 2.6. Functional Data Analysis

Functional data analysis (FDA) is a statistical framework for analyzing data that take the form of functions, such as time series or other continuous processes [26]. Unlike traditional approaches that treat observations as discrete points, FDA represents each time series as a smooth, continuous curve. This enables the analysis of overall trajectories, derivatives, and temporal patterns while accounting for measurement noise and irregular sampling intervals [11]. FDA has been applied in a range of fields—including neuroscience, biomechanics, and finance—where understanding and comparing dynamic processes is essential. By leveraging the smoothness and continuity of functional data, FDA provides a robust method for detecting meaningful differences between time-varying signals [27].

In this study, FDA was implemented using R (version 4.2.2) within RStudio (version 2024.12.0+467; Boston, MA, USA). The open-source fda package (version 6.1.8; Ramsay and Silverman, 2005 [28]) was used for analysis. For each dichotomous grouping variable, the observed test statistic curve, F^2^(*t*), was computed and plotted. This statistic reflects, at each time point, *t*, the squared difference between the group mean curves relative to the within-group variance. Larger spikes in the F^2^(*t*) curve indicate greater divergence between group means at that specific time. Statistical significance was determined at a threshold of *p* < 0.05 using the F^2^(*t*) curve. To maximize clinical relevance and reduce the risk of false positives due to transient signal artifacts, a finding was considered statistically significant only if the test statistic exceeded the critical threshold for at least 5 consecutive seconds post-stand.

## 3. Results

A total of 25 community-dwelling women were included in the study, with a mean age of 70.3 years (range: 59–82). Participants were dichotomized into an older group (n = 14, age ≥ 70 years) and a younger group (n = 11, age < 70 years). IOH was present in 14 participants, while 11 did not meet the criteria for IOH. Based on BMI, 15 participants were classified as overweight or obese (BMI ≥ 25 kg/m^2^, range: 25–36), and 10 were considered non-overweight (BMI 17 to <25 kg/m^2^). Regarding medication use, 8 participants reported current use of antihypertensive medications, while 17 were not taking any such medications. Physical frailty was assessed using the continuous SHARE-FI score; 12 participants had a score < −0.5 (classified as less frail), while 13 scored ≥−0.5 and were considered to have higher frailty.

**Figure 1:**
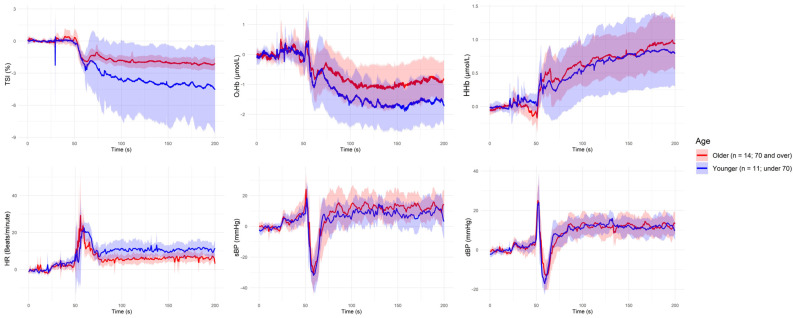
Group-level mean trajectories with 95% confidence intervals for neurovascular and cardiovascular signals during the active stand test, stratified by age group. Participants were classified as younger (<70 years, n = 11; blue line) or older (≥70 years, n = 14; red line). The active stand was initiated at 50 s. Signals shown include tissue saturation index (TSI), oxygenated hemoglobin (O_2_Hb), deoxygenated hemoglobin (HHb), heart rate (HR), systolic blood pressure (sBP), and diastolic blood pressure (dBP).

**Figure 2:**
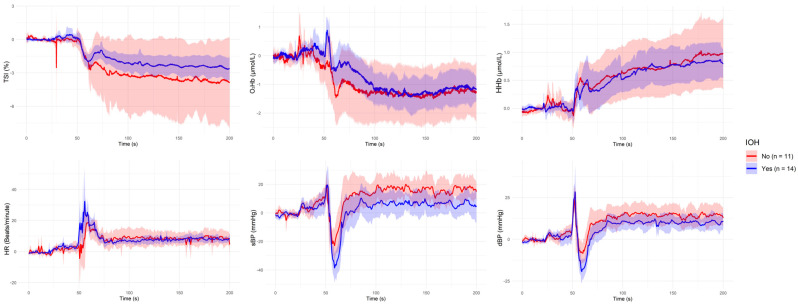
Group-level mean trajectories with 95% confidence intervals for neurovascular and cardiovascular signals during the active stand test, stratified by the presence of initial orthostatic hypotension (IOH). Participants were classified as IOH-positive (n = 14; blue line) or IOH-negative (n = 11; red line) based on standard hemodynamic criteria. The active stand was initiated at 50 s. Signals shown include tissue saturation index (TSI), oxygenated hemoglobin (O_2_Hb), deoxygenated hemoglobin (HHb), heart rate (HR), systolic blood pressure (sBP), and diastolic blood pressure (dBP).

**Figure 3:**
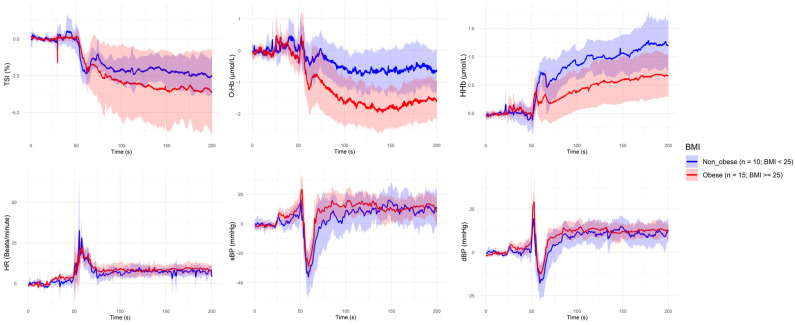
Group-level mean trajectories with 95% confidence intervals for neurovascular and cardiovascular signals during the active stand test, stratified by body mass index (BMI). Participants were classified as non-overweight (BMI < 25 kg/m^2^, n = 10; blue line) or overweight/obese (BMI ≥ 25 kg/m^2^, n = 15; red line). The active stand was initiated at 50 s. Signals shown include tissue saturation index (TSI), oxygenated hemoglobin (O_2_Hb), deoxygenated hemoglobin (HHb), heart rate (HR), systolic blood pressure (sBP), and diastolic blood pressure (dBP).

**Figure 4:**
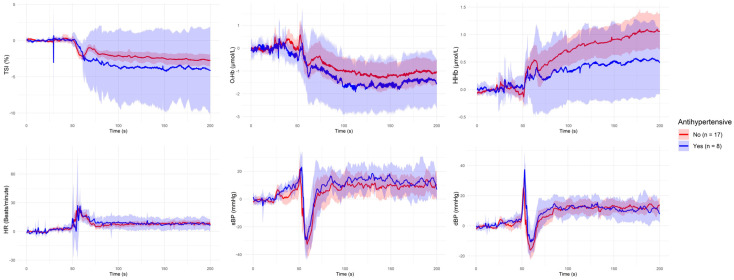
Group-level mean trajectories with 95% confidence intervals for neurovascular and cardiovascular signals during the active stand test, stratified by antihypertensive medication use. Participants were categorized as not using antihypertensive medication (n = 17; red line) or currently using antihypertensive medication (n = 8; blue line). The active stand was initiated at 50 s. Signals shown include tissue saturation index (TSI), oxygenated hemoglobin (O_2_Hb), deoxygenated hemoglobin (HHb), heart rate (HR), systolic blood pressure (sBP), and diastolic blood pressure (dBP).

**Figure 5:**
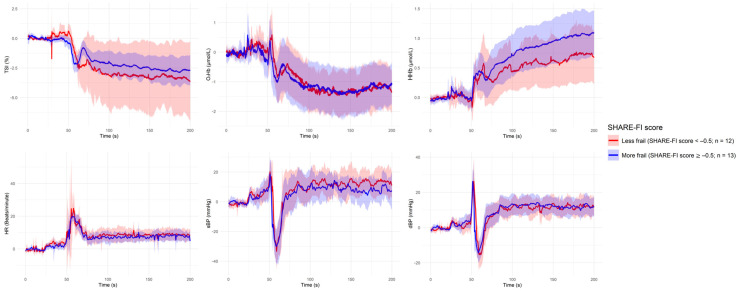
Group-level mean trajectories with 95% confidence intervals for neurovascular and cardiovascular signals during the active stand test, stratified by physical frailty status. Participants were classified as less frail (SHARE-FI score < −0.5; n = 12; red curve) or more frail (SHARE-FI score ≥ −0.5; n = 13; blue curve). The active stand was initiated at 50 s. Signals shown include tissue saturation index (TSI), oxygenated hemoglobin (O_2_Hb), deoxygenated hemoglobin (HHb), heart rate (HR), systolic blood pressure (sBP), and diastolic blood pressure (dBP).

Figure 1, Figure 2, Figure 3, Figure 4 and Figure 5 present the group-level time series plots of six physiological signals—TSI, O_2_Hb, HHb, HR, sBP, and dBP—during the active stand test, stratified by the characteristics of interest. Each plot displays mean trajectories with 95% confidence intervals for two comparison groups covering a 200 s window (0–200 s), with the stand occurring at 50 s. Specifically, Figure 1 compares responses between younger (<70 years) and older (≥70 years) participants; Figure 2 compares those with and without IOH; Figure 3 compares non-overweight (BMI < 25 kg/m^2^) and overweight/obese (BMI ≥ 25 kg/m^2^) participants; Figure 4 contrasts participants based on antihypertensive medication use; and Figure 5 shows trajectories by physical frailty status based on median SHARE-FI scores.

**Figure 6:**
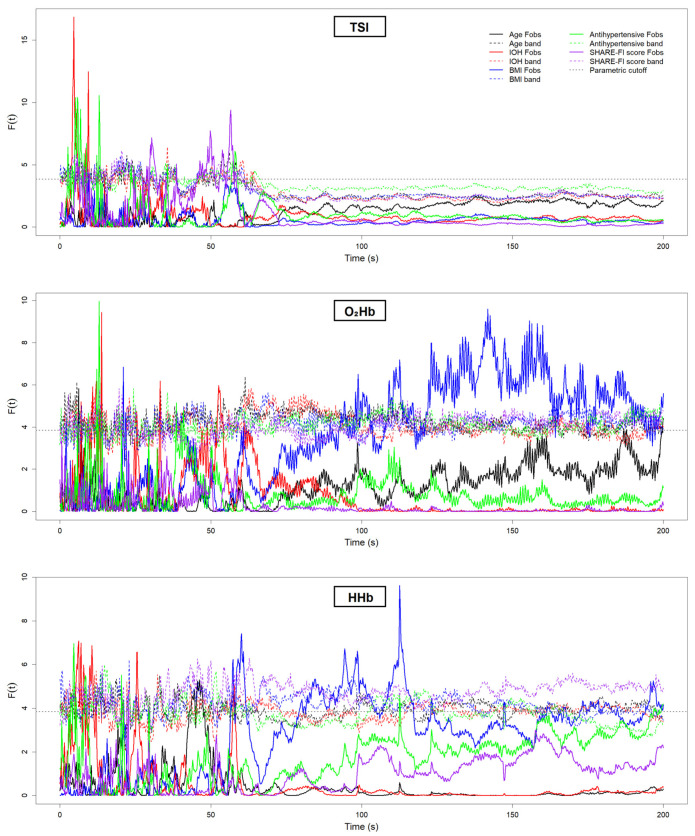
Functional data analysis (FDA) F^2^(*t*) statistic curves for neurovascular signals—tissue saturation index (TSI), oxygenated hemoglobin (O_2_Hb), and deoxygenated hemoglobin (HHb)—measured during the 200 s active stand test. Each solid colored line represents the observed F^2^(*t*) statistic for a specific group comparison (age, IOH, BMI, antihypertensive use, and frailty). The corresponding dashed colored lines indicate the permutation-derived critical thresholds for statistical significance (*p* < 0.05). The dotted gray horizontal line represents the conventional 95% parametric F cutoff, derived from the F-distribution under assumptions of normality and equal variances, and is included for reference. Periods where the observed F^2^(*t*) curve exceeds its permutation-derived threshold for at least 5 consecutive seconds are interpreted as clinically significant differences in signal trajectories between groups. The active stand began at 50 s. In the legend, “Fobs” denotes the observed F^2^(*t*) statistics, and “band” refers to the permutation-derived significance threshold for each group comparison.

**Figure 7:**
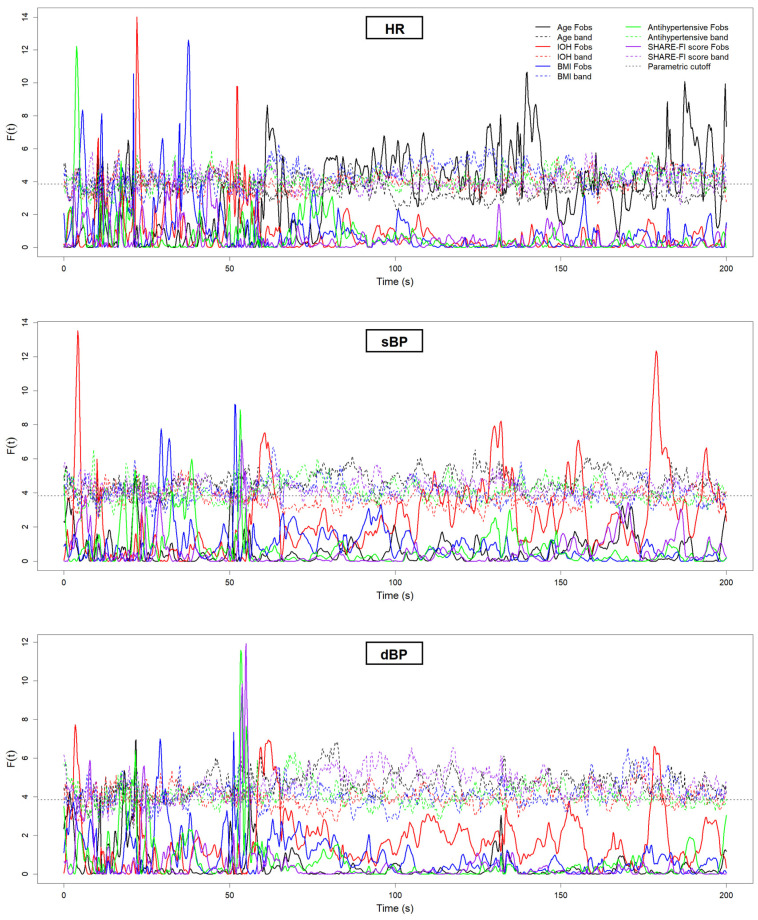
Functional data analysis (FDA) F^2^(*t*) statistic curves for cardiovascular signals—heart rate (HR), systolic blood pressure (sBP), and diastolic blood pressure (dBP)—measured during the 200 s active stand test. Each solid colored line represents the observed F^2^(*t*) statistic for a specific group comparison (age, IOH, BMI, antihypertensive use, and frailty). The corresponding dashed colored lines indicate the permutation-derived critical thresholds for statistical significance (*p* < 0.05). The dotted gray horizontal line represents the conventional 95% parametric F cutoff, derived from the F-distribution under assumptions of normality and equal variances, and is included for reference. Periods where the observed F^2^(*t*) curve exceeds its permutation-derived threshold for at least 5 consecutive seconds are interpreted as clinically significant differences in signal trajectories between groups. The active stand began at 50 s. In the legend, “Fobs” denotes the observed F^2^(*t*) statistics, and “band” refers to the permutation-derived significance threshold for each group comparison.

Figure 6 and Figure 7 present the results of the FDA conducted on the neurovascular (Figure 6) and cardiovascular (Figure 7) signals. Each plot displays the F^2^(*t*) test statistic curve for the relevant grouping variables, illustrating the moments in time where statistically significant differences between groups emerged. Peaks that exceed the permutation-derived significance threshold (dotted line) indicate time intervals of meaningful group-level divergence. Figure 6 focuses on the NIRS-derived signals, while Figure 7 shows the results for the cardiovascular signals. Table 1 provides a summary of these significant findings, including the direction of differences and the approximate time periods post-stand where group-level divergences lasting at least 5 consecutive seconds were observed.

## 4. Discussion

This pilot study, conducted using a sample of older women, aimed to examine whether dichotomous participant characteristics—related to age, IOH, BMI, antihypertensive medication use, and physical frailty status—were associated with differential neurocardiovascular responses to an active stand test. Functional data analysis (FDA) was employed to characterize the timing and nature of postural adaptation patterns beyond conventional time point comparisons. Group-level differences revealed that younger participants (<70 years) exhibited significantly higher HR in multiple periods following the stand (~10 s, ~30 s, ~90 s, and ~140 s post-stand) compared to their older counterparts. Participants with IOH demonstrated significantly lower sBP at ~10 s, ~80 s, and ~130 s post-stand and lower dBP at ~10 s post-stand. Among participants classified as overweight/obese (BMI ≥ 25 kg/m^2^), significantly lower levels of HHb were observed at ~10 s, ~30–50 s, and ~60 s post-stand, while O_2_Hb levels were reduced at ~50 s, ~60 s, ~70–110 s, ~130 s, and ~140 s post-stand. No statistically significant group-level differences were observed based on antihypertensive medication use or frailty status.

The observed group-level differences in HR and blood pressure following the orthostatic challenge are consistent with established physiological mechanisms and the prior literature. The finding that younger participants exhibited greater post-stand HR elevations aligns with well-documented age-related declines in baroreflex sensitivity and autonomic responsiveness, which contribute to a diminished chronotropic response in older adults [6,29,30,31]. Similarly, the lower sBP and dBP observed among participants with IOH are expected and consistent with the diagnostic criteria for IOH—particularly around 10 s post-stand—as well as previously published findings [7].

These expected and internally consistent findings support the validity of the BMI-related results and reduce the likelihood that they are spurious. Notably, participants with a BMI ≥ 25 kg/m^2^ exhibited significantly lower levels of O_2_Hb and HHb in the frontal cortex following the orthostatic challenge, suggesting impaired cerebral oxygenation in individuals with excess adiposity. This observation is in line with work by Knight et al. (2021), who demonstrated that higher BMI, waist circumference (WC), and waist-to-hip ratio (WHR) were each associated with lower cerebral blood flow in older adults, as measured by arterial spin labeling MRI. Their analysis further highlighted that a 1 cm increase in waist circumference had an equivalent impact on cerebral perfusion as one additional year of aging, underscoring the vascular burden of central adiposity. Importantly, they also found that high levels of physical activity moderated the negative associations between obesity metrics and cerebral blood flow. Taken together, these findings reinforce the plausibility of our NIRS-based observations and point to a physiologically grounded mechanism whereby increased adiposity impairs neurovascular function [32].

In contrast, no statistically significant differences were observed based on antihypertensive medication use or frailty status. The absence of medication-related effects may reflect the heterogeneity of antihypertensive drug classes represented in the sample or the varying influence of these agents on autonomic and vascular tone. Similarly, the binary classification of frailty using the SHARE-FI threshold may have lacked the sensitivity to detect more nuanced differences in physiological reserve, particularly within this relatively high-functioning cohort. Indeed, according to the original SHARE-FI classification [14], only four participants met the criteria for pre-frailty, while the remainder were classified as non-frail. Although the use of a median split allowed for the construction of two approximately equal-sized comparison groups, it may not have captured a clinically meaningful distinction in physical frailty status in a sample composed predominantly of robust individuals.

Although this finding did not reach statistical significance in our analysis, the observation that younger adults exhibited a larger post-stand drop in O_2_Hb—remaining lower during the recovery phase compared to older participants—has also been noted by Klop et al. in small samples of both community-dwelling individuals and geriatric outpatients, although it was not formally tested for statistical significance in their work [33]. In addition, Klop et al. reported that cerebral oxygenation recovery values were lower in participants with orthostatic hypotension (OH) and that those who experienced symptomatic OH exhibited a deeper maximum O_2_Hb drop than those with asymptomatic responses [34]; however, in their study, OH was defined as a drop in systolic BP of ≥20 mmHg and/or diastolic BP of ≥10 mmHg occurring 1 to 3 min after standing, based on a 5 s moving average—consistent with the classical OH definition [35], not initial IOH. Notably, none of our included participants met the criteria for classical OH. In relatively healthy individuals, IOH has been associated with faster standing speeds and may reflect effective compensatory mechanisms rather than pathological dysregulation [36,37,38]. It is, therefore, possible that the suggested trend toward higher O_2_Hb levels at 10–20 s post-stand in our sample represents a compensatory response in otherwise healthy individuals adapting to a transient BP drop. This potential relationship warrants further investigation in larger, well-characterized cohorts. Furthermore, although not statistically significant in our analysis, there was a possible trend toward an exaggerated orthostatic sBP pressor response in participants with elevated BMI. This is consistent with prior evidence linking obesity and related conditions—such as diabetes, hypertension, and aging—with exaggerated orthostatic pressor responses (ERTSs) or orthostatic hypertension (OHT), likely due to heightened sympathetic activity and baroreflex dysregulation [39]. Again, further investigation in larger samples is warranted.

Several methodological considerations merit acknowledgment. First, our modest sample size limited statistical power, particularly for subgroup comparisons. Although FDA improves sensitivity to time-localized effects in small samples, it remains susceptible to signal noise and individual variability—especially when multiple physiological signals are analyzed concurrently. Second, the study cohort was restricted to community-dwelling older women, which limits the generalizability of the findings to men and to more frail or institutionalized populations. Third, while NIRS provides a valuable, non-invasive proxy of cortical oxygenation, it is influenced by extracranial blood flow and does not offer a direct measure of cerebral perfusion. Additionally, the cross-sectional observational design precludes causal inference and limits conclusions about the long-term clinical implications of the observed responses.

Furthermore, although continuous physiological monitoring provides high temporal resolution, such data are vulnerable to motion-related and technical artifacts—particularly around the transition from supine to standing. In this study, transient fluctuations were observed both pre- and post-stand, which may reflect artifacts rather than true physiological changes. To mitigate this, a conservative approach was adopted: only group differences that statistically persisted for at least five consecutive seconds were considered clinically meaningful. While this criterion helped reduce the influence of brief, potentially spurious spikes, it may also have excluded short-duration responses of physiological relevance. Future work should incorporate automated artifact detection algorithms and larger, more diverse cohorts to validate and extend these exploratory findings.

Despite this study’s limitations, the findings support the feasibility and utility of FDA for evaluating continuous physiological responses under clinically relevant testing conditions. FDA enables time-resolved analysis of complete signal trajectories, providing a valuable alternative to conventional methods—especially in studies with limited sample sizes or exploratory aims. The results indicate that elevated BMI, older age, and the presence of IOH may be associated with distinct neurocardiovascular response patterns during orthostatic challenge. These physiological differences may help explain increased susceptibility to falls, syncope, and cognitive impairment in older adults, particularly among those older and with higher adiposity. However, further research involving larger, more diverse populations and longitudinal follow-up is needed to validate these observations and clarify their clinical relevance.

## Figures and Tables

**Table 1:** Summary of statistically significant group-level differences in neurocardiovascular responses during the active stand test as identified by functional data analysis (FDA). Each row represents a dichotomous grouping variable (e.g., age, IOH status), and the corresponding physiological signal(s) where significant differences were observed. The “Direction of Difference” column indicates which groups had lower values, and the “Significant Period (s)” column reports approximate time intervals post-stand (in seconds) during which the difference exceeded the permutation-derived threshold for at least 5 consecutive seconds.

Grouping Criterion	Signal	Direction of Difference (Group with Lower Value)	Significant Period (s)
Younger (<70 years)	HR	Aged (≥70 years) < Younger	~60 s, ~80 s, ~140 s, ~190 s
IOH: Yes	sBP	IOH < Non-IOH	~60 s, ~130 s, ~180 s
IOH: Yes	dBP	IOH < Non-IOH	~60 s
Overweight (BMI ≥ 25 kg/m^2^)	HHb	Overweight < Non-overweight	~60 s, ~90–100 s, ~110 s
Overweight (BMI ≥ 25 kg/m^2^)	O_2_Hb	Overweight < Non-overweight	~100 s, ~110 s, ~120–160 s, ~180 s
Antihypertensive Use: Yes	None	—	—
Frailty (SHARE-FI ≥ −0.5)	None	—	—

## Data Availability

The data presented in this study are available upon reasonable request from the corresponding author. The data are not publicly available due to ethical and privacy restrictions, as they contain identifiable information collected from human participants.
